# The effect of side-alternating vibration therapy on mobility and health outcomes in young children with mild to moderate cerebral palsy: design and rationale for the randomized controlled study

**DOI:** 10.1186/s12887-020-02377-2

**Published:** 2020-11-05

**Authors:** Alena Adaikina, Paul L. Hofman, Silmara Gusso

**Affiliations:** 1 Liggins Institute, University of Auckland, Auckland, New Zealand; 2 Department of Exercise Sciences, University of Auckland, Auckland, New Zealand

**Keywords:** Cerebral palsy, Vibration therapy, Bone density, Mobility, Gross motor function, Body composition, Muscle function, Respiratory function, Quality-of-life

## Abstract

**Background:**

Cerebral palsy (CP) is the most common cause of physical disability in early childhood. Vibration therapy (VT) is a promising rehabilitation approach for children with CP with potential to impact mobility, bone and muscle health as demonstrated by extant research. However, it is still unclear how long therapy must be conducted for and what the optimal vibration frequency is in order to gain health benefits.

**Methods/design:**

The study is a randomized clinical trial evaluating and comparing the effects of two vibration frequency (20 Hz vs 25 Hz) and duration protocols (12 weeks vs 20 weeks) of side-alternating VT on mobility and other health parameters in children with CP. Children aged 5–12 years old with CP and GMFCS level I-III who are able to understand instruction and safely stand are eligible for the study. Exclusion criteria include bone fracture within 12 weeks of enrolment; acute conditions; the history of significant organic disease; the history of taking anabolic agents, glucocorticoids, growth hormone, and botulinum toxin injection into lower limbs within 3 months of enrolment. All participants will act as their own control with a 12-week lead-in period prior to intervention. The intervention period will consist of 20 weeks of home- or school-based VT 9 min per day, 4 times a week. After the baseline assessment, participants will be randomized to either a 20 Hz or 25 Hz vibration-frequency group. The primary outcome is mobility measured by a 6-min walking test, with analysis performed on the principle of intention to treat. Secondary outcomes include body composition, muscle strength, physical activity level, balance, gross motor function, respiratory function, and quality of life. Participants will undergo four assessment visits over the study period: baseline, at weeks 12, 24, and 32.

**Discussion:**

The results of the study will provide evidence-based insights into the health benefits of side-alternating VT as a therapeutic tool in young children with cerebral palsy. The investigation of different vibration training protocols will help define the optimal parameters of intervention protocols (duration, frequency) of side-alternating VT to maximize outcomes on the health of 5–12-year-old children with CP.

**Trial registration:**

Australian New Zealand Clinical Trials Registry (ANZCTR): 12618002026202 (Registration date 18/12/2018).

## Background

Cerebral palsy (CP) is a group of disorders of the development of movement and posture, causing activity limitation attributed to non-progressive disturbances that occurred in the developing fetal or infant brain [1] with the estimated prevalence of 2 per 1000 children [2, 3]. The most common characteristic of CP is a motor impairment that embraces reduced mobility, impaired motor skills, changes in muscle tone and strength, restricted range of motion, and deterioration of balance and gait [4]. Improving mobility and functional activity is one of the main therapeutic goals for children with CP [5]. Given the key factors contributing to impaired mobility are muscle weakness and spasticity [6–8], current therapies of children with CP include orthopedic surgery, antispastic drug therapy, and a variety of muscle strength rehabilitation tools [9, 10]. However, these interventions are time- and cost-consuming, complex, and require specialized equipment and/or professional staff [11–13].

Vibration therapy (VT) was introduced about 20 years ago as a prospective rehabilitation approach for children and adults with CP. VT is well tolerated by children with CP with no serious adverse effects reported [14–18]. The most frequently described side effects include temporary redness and itchiness of the leg skin that has an adaptive effect [17, 19, 20]. To date, only one study has reported fatigue and pain as a reason of discontinuation single vibration sessions in fewer than 1% of training sessions with no evidence that these effects had been caused by vibration training [21]. VT has been found to be an effective and cost-efficient method to improve muscle strength [15, 17], decrease spasticity [17] and, consequently, improve mobility [16, 17, 21] in this population. In addition, previous studies have reported VT to be effective in improving muscle function [16, 17, 22], bone mineral density [16, 22, 23], gross motor function [17, 22], as well as the quality of life [16] in children and young adults population. A recent review by Ritzmann et al. (2018) that appraised 28 published studies on the effects of VT on functional, neuromuscular and structural parameters of patients with CP revealed an emerging collective evidence that speaks to the potential of VT in ameliorating the symptoms of the disease in both short term (i.e., reducing reflex excitability, spasticity, and coordination deficits) and long term (i.e., improving movement ability, increasing muscle mass and bone mineral density) [24]. However, despite the promising results demonstrated by VT, its wider application is limited by the heterogeneity of methodological approaches used in extant research, with protocols varying in frequency and duration, which complicates the interpretation of the results and the development of treatment protocols [15, 17, 21–23, 25, 26]. In the reported longitudinal studies, the duration of the vibration training varied from 3 to 24 weeks and the target frequency of the vibration stimuli ranged from 18 to 25 Hz [24]. To our knowledge, there have been no published studies that compared the effect of different duration and frequency parameters of side-alternating vibration therapy on the health outcomes of children with CP in longitudinal studies. Therefore, it is still unclear whether higher intensities and/ or longer interventions will bring more health benefits or not. As summarized in the aforementioned systematic review of extant research on the effectiveness of VT, “while the success of VT has been outlined clearly … systematic judgment on the choice of VT parameters requires further investigation” ([24], p. 1623).

Available studies have also reported a diverse range of outcomes [15, 17, 22, 23, 25, 26], which complicates the comparison of the available data across studies. To date, the only study with a comprehensive set of assessment has been published by Gusso et al. (2016) and involved adolescents and young adults with cerebral palsy. The study found a positive effect of 20 weeks of side-alternating VT on mobility, muscle mass and function, bone mineral density as well as the quality of life [16]. The findings were, however, limited to the adolescent population and cannot be generalized to younger children due to the known impact of age, growth speed, puberty and sex hormone status on the muscle and bone health, development, and mobility level [27–30]. Therefore, in designing the study and selecting the assessment tools, we sought to conduct a robust assessment that would provide a comprehensive insight into the effectiveness of VT on the health of children 5–12 years old with CP (i.e., muscle function, muscle strength (isometric and dynamic), and bone health) and to evaluate the effect of different parameters of VT on this population.

Apart from the musculoskeletal involvement, children with CP may present with multiple medical conditions, including compromised respiratory function, which affects the quality of life, hospitalization frequency, and life expectancy [31–34]. As shown in previous research, respiratory muscle weakness and poor cough both contribute to respiratory impairment in children with CP [35–37]. To our knowledge, no studies have explored the impact of VT on respiratory function in children with CP. Available studies were conducted in the adult population with chronic obstructive pulmonary disease [38, 39]. We hypothesize that VT has the potential to improve respiratory function by improving the strength of diaphragm and core muscles which play an important role in the breathing cycle and respiratory function [37, 40, 41].

Therefore, our study proposed to examine and compare the effect of two frequency and duration protocols of side-alternating vibration therapy on mobility, muscle and bone health, motor function, as well as respiratory function and well-being in young children 5–12 years of age with mild to moderate CP.

## Methods

### Study design

**Fig. 1:**
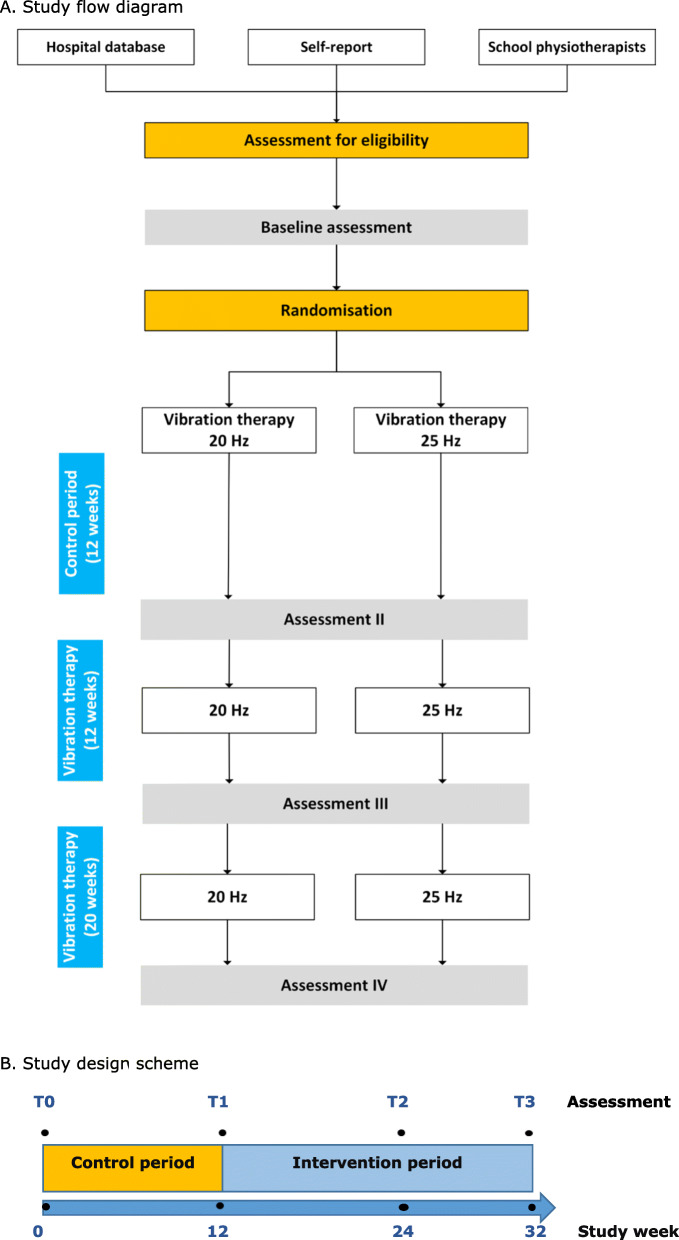
Study design. **a** Study flow diagram. **b** Study design scheme

The study is designed as a randomized clinical study, where all participants will act as their own control, with a 12-week lead-in period prior to the intervention (Fig. 1). For the intervention part of the study, participants will be randomized into two groups varying in the frequency of vibration stimuli. The study was approved by the Health and Disability Ethics Committee (Ministry of Health, New Zealand; 19/NTB/2), and was registered with the Australian New Zealand Clinical Trials Registry (ANZCTR: 12618002026202). Institutional approval has been obtained from the Auckland District Health Board (ADHB).

### Participants

Children aged 5–12 years with any type of cerebral palsy and a GMFCS classification level I-III (very mild to moderate) are eligible for the study. Participants need to be able to understand and follow protocol instructions and safely stand on the vibration platform. Exclusion criteria include a bone fracture within 12 weeks of enrolment; acute thrombosis, tendinitis, nephrolithiasis, discopathy or arthritis; history of clinically significant organic disease; history of taking anabolic agents, glucocorticoids or growth hormone for at least 1 month within 3 months of enrolment; and a history of botulinum toxin injection into lower limbs within 3 months of enrolment.

### Recruitment

Potential participants will be identified from the ADHB database using the described exclusion/inclusion criteria (Fig. 1a). Those who fit the eligibility criteria will initially be approached by pediatricians, nurses or physiotherapists with a short explanation and flyer of the study. School physiotherapists at satellite schools in the Auckland area will also be notified about the study and will be able to inform prospective participants. Self-referrals will also be accepted. Once parents/caregivers of a potential participant have indicated an interest in participating, they will be contacted by a member of the research team to provide a detailed explanation concerning the study design, assessment tests, and vibration therapy. A participant information sheet will then be sent by email or regular mail. Two-five days later, the researchers will contact prospective families to answer any questions, and if a family agrees to participate, the initial appointment will then be scheduled.

### Randomization

Randomization will be performed immediately after the initial assessment using an online random number generator available at https://www.randomizer.org. Participants will be allocated to either group 1 (VT frequency 20 Hz) or group 2 (VT frequency 25 Hz). The same member of the research team will enrol participants and assign them to the intervention group. Given that the current study is part of a PhD project, all the procedures (i.e., randomisation, testing, VT sessions) will thus be run by the same member of the research team, making the blinding of the researchers unfeasible in this study. Additionally, the vibration frequencies produced by the Galileo™ vibration platform are readily observable by both researchers and study participants, which makes it unfeasible to ensure blinding not only at the randomization but the intervention stage as well.

### Control period

The control period will start immediately after the first assessment visit and will last 12 weeks (Fig. 1a). During the control period, participants will continue their usual activities and care.

### Intervention

Following the control period, a 20-week intervention period will start. It will consist of 4 vibration sessions a week performed on Galileo Basic vibration plates (Novotec Medical, Pforzheim, Germany). Participants will start from three 1-min bouts of vibration training at the frequency of 12 Hz with a gradual increase of both frequency and duration over the first 4 weeks to achieve the target frequency and time (3 sets of 3 min vibration training alternating with 3-min breaks between them). The target frequency of the vibration will vary depending on the intervention group (either 20 Hz or 25 Hz) and will be maintained for the remainder of the intervention period. Other parameters will be kept comparable in both treatment groups: amplitude – 2-4 mm, acceleration – maximum 11.4 g. Participants will stand barefoot on the plate with knees slightly bent, back straight, and arms free. For participants with poor balance, an adjustable metal frame will be used for safety purposes.

The vibration therapy frequencies (20 Hz vs 25 Hz) have been selected based on current literature and the Galileo vibration platforms manufacturing specifications. According to the latter, the main effect of low frequencies (12–20 Hz) lies in an improvement of muscle function and coordination whereas high frequencies (more than 20 Hz) have an impact on muscle power and muscle force [42]. According to the literature, 20 Hz is a typically recommended frequency for children with cerebral palsy [43]. In addition, 20 Hz has been shown to be the safest of low frequencies due to low vibration transmissibility to the head [19] and better coordination and posture stabilisation during the vibration training sessions [44]. 25 Hz was chosen as it is the highest frequency that has been used in long-term studies of side-alternating vibration training that involved children with cerebral palsy and that has demonstrated no severe adverse effects [24]. To summarize, the two frequencies were selected since they provide the optimal combination of expected results, as informed by extant literature and equipment specifications, and adherence to the ethical obligation of not compromising the patients’ safety.

Families have two options for where to perform the training: either at home or at a child’s school. In the case of home-based VT, parents/caregivers will have an instruction session at the Liggins Institute at the end of the second assessment visit on how to perform VT and provide supervision of home sessions. In order to monitor progress and provide feedback/support, a member of the research team will supervise home-based VT once every 2 weeks for the first 8 weeks and once a month for the remaining 12 weeks. In addition, parents/caregivers will be contacted by phone/ email for the follow-up. Vibration therapy at schools will be supervised by a member of the research team. Before the team member enters the school, an agreement form will be signed by the school’s principal/deputy principal to allow access. Parents of children who are performing vibration therapy at school sites will be followed up with in person (e.g. during assessment visits) as well as by phone and/or email.

Participants will be provided a VT diary with specifications of the frequency and duration of VT for each training session and will be asked to record the completed sessions, reasons for skipping sessions and comments regarding any adverse events.

### Primary outcome

The primary outcome measure is mobility, which will be assessed by the distance walked in the 6-min walk test (6MWT) at four-time points: baseline, in 12 weeks (the end of the control period), after 12 weeks of vibration therapy, and after completing 20 weeks of vibration therapy (Fig. 1b).

### Secondary outcomes

The secondary outcomes include body composition, muscle power, physical activity level, balance, gross motor function, respiratory function, and quality of life. All the outcomes will be assessed four times over the study period: baseline, at 12, 24, and 32 weeks (Fig. 1b).

### Other aims and outcomes

Participants’ fall history will be obtained using parents/caregivers recalls/reports of falls frequency and reasons during the study period by completing a falls diary.

### Clinical assessments/data collection

All participants will have four assessment visits at the Maurice and Agnes Paykel Clinical Research Unit (Liggins Institute, The University of Auckland) (Fig. 1b): 1 – baseline (T0), 2–12 weeks after the lead-in period (control) (T1), 3 – after 12 weeks of vibration therapy (T2), 4 – after completing 20 weeks of vibration therapy (T3). During the first visit, written informed consent will be obtained from a participant’s parent/guardian and, if possible, a child assent form from participants older than 7 years old. Information on demographic, ethnicity, medical history (including the history of birth, comorbidities, previous surgery, Botox injections, injuries, and admissions to the hospital) and medications currently taken will be recorded. We also will collect information on the frequency and duration of activities children undergo at school (e.g., physical education classes) and out of school (e.g., swimming, horse riding, walking with a dog, etc.). The procedures will be similar for all four visits and will also include anthropometric measurements, resting blood pressure and heart rate, mobility, body composition, muscle function, physical activity, gross motor function and respiratory function assessments, and the quality-of-life questionnaire.

### Anthropometric data

Height will be measured using a wall-fixed Harpenden Stadiometer (Holtain Ltd., Crymych, UK) three times, and the average of the three will be recorded to the nearest 0.1 cm. Weight will be measured on the calibrated electronic scale (Wedderburn WM206., NZ) without shoes and outerwear, when possible, to the nearest 0.1 kg. Based on the height and weight data, the Body mass index will be calculated electronically, using an online calculator available at https://www.heartfoundation.org.nz and recorded in kg/m^2^.

### Blood pressure, heart rate, and oxygen saturation

Blood pressure, heart rate, and oxygen saturation will be measured on the dominant arm in a sitting position after 10 min of rest prior to the start of functional tests. We will use a standard manual mercury sphygmomanometer with an appropriately-sized cuff to measure blood pressure and a finger sensor to record oxygen saturation and heart rate (Dinamap ProCare 100, GE Healthcare, Freiburg, Germany).

### Mobility

Two tests, a 6MWT and a 10-m walk test (10MWT), will be used to assess mobility. The 6MWT has been shown to be safe, easy to perform, reliable and valid in children with CP [45] and has been widely used in clinical trials in this population [16, 17, 46]. For the 6MWT, participants will be asked to walk as fast as they can between 2 cones separated by 25 m along a long, flat, straight indoor corridor for 6 min [47]. They will be able to stop and rest if required, and then continue walking until the 6 min are up. During the test, children will be closely supervised by an assessor and will be given standardized 6MWT instructions and encouragement [45, 48]. The total distance covered will be recorded to the nearest 0.5 m, along with the time taken to reach individual milestones (25 m). The participant’s perceived exhaustion will be assessed by using the OMNI-walk/run RPE scale before and after the test [49]. The test will be performed with shoes on; children may use their walking aid or orthotics, but not a wheelchair, to complete the test. For the 10MWT, a child will be instructed to walk as fast as (s) he can. The time to cover 6 m will be recorded, with two meters given for acceleration and two meters for deceleration. The test will be performed three times; the best time to the nearest hundredth of a second will be taken to calculate the walking speed (meters/sec). Verbal encouragement will be given during the walking task. The same assessor will be timing, with a stopwatch, all the participants to minimize the effect of human errors on the outcome. For both tests, if walking aids and orthotics are used, a note will be made in the record, and they will be consistently used for all 4 visits assessments.

### Muscle function status

Muscle function status will be measured by a number of assessments. Muscle strength will be measured with a hand-held dynamometer (MicroFET2, Hoggan Scientific, USA). Hand-held dynamometry has been reported as a reliable and valid measurement tool to assess muscle strength in clinical settings [50, 51]. Five muscle groups of the lower extremities (hip flexors and extensors, knee flexors and extensors, and ankle dorsiflexors) will be assessed using a “make”-technique [50]. The test will be performed by the same examiner, with the testing sequence kept in the same order at all the assessments. Standard instructions of “push as hard as you can” will be given to participants for each trial; consistent verbal encouragement will be given to children during timed, 3-s contraction period for all trials. Measurements will be performed three times for each muscle group with 30 s interval rest between assessments. The average of the three measurements will be taken for analysis.

The Leonardo™ Mechanography Ground Reaction Force Plate (Novotec Medical, Pforzheim, Germany) will be used to assess dynamic muscle function by chair rising test (CRT) and single two-leg jump test (S2LJT) [52]. The Force Plate has been reported as a reliable and valid instrument in children with CP [53]. The CRT test will be performed with a specially designed seat (Novotec Medical, Pforzheim, Germany) placed on the plate, with participants standing up and sitting down 5 times in a row as fast as possible with arms on chest. The test will be performed three times, with the best result recorded. For analysis, the total time to complete the test, maximum force and power in both legs will be used. For S2LJT, participants will be instructed to jump as high as possible using both legs and landing on the forefoot. The test will be performed three times after one practice, with the best result recorded. Variables of interest include the maximal jump height (m), the maximal total power per body weight (W/kg), maximal total force (kN), maximal velocity (m/s), and the Esslinger Fitness Index.

### Balance

The balance will be tested using the Leonardo™ Mechanography Ground Reaction Force Plate (Novotec Medical, Pforzheim, Germany) with the participant standing still with free arms for 10 s in three different positions: on both legs, on the right, and on the left leg (if a child can balance on one leg). The best result of the three trials will be recorded. The outcome parameter of the test is the standard ellipse area (cm^2^), which identifies postural stability [54].

### Gross motor function measure

Gross motor function measure (GMFM) is a clinical tool designed to evaluate changes in gross motor function in children with cerebral palsy both in clinical practice and for research purposes [55, 56]. It has demonstrated good test-retest reliability and responsiveness [57], and high construct validity [58, 59]. We will assess two dimensions from the original 88-item measure (GMFM-88): D (standing) and E (walking, running, jumping) [56]. The test will be performed in a playroom of the Clinical research unit at the Liggins Institute by the same research team member.

### Respiratory function

The respiratory function will be tested using a CareFusion MicroLab MK8 spirometer (Care Fusion; Chatham, Kent, UK). Before each measurement, the spirometer will be calibrated. Participants will be rested for 10 min before measurements. Before starting the test, subjects will be instructed on the test procedure, which will then be demonstrated by a researcher. A nose clip will be used during the test. Participants will perform a maximum forced inhalation (instructions: “Breath in as hard as you can”), followed by a powerful, quick, forced expiration (instructions: “Breath out as fast and as hard as you can”). At least three technically appropriate measurements will be performed, with the best result taken for statistical analysis. Measures will include forced vital capacity (FVC) (L), forced expiratory volume in 1 s (FEV1) (L), and ratio FEV1/FVC.

### Body composition

Dual-energy X-ray absorptiometry (DXA) (Lunar Prodigy 2000, General Electric, Madison, WI, USA) will be used to determine body composition, including lean mass, percentage fat, bone mineral density (BMD) and bone mineral content (BMC). DXA is a safe, standardized instrument to measure bone density both in clinical settings and for research purposes [60]. A calibration check of the DXA machine will be performed on the day prior to starting measurements. We will perform two scans on each participant: total body and lumbar spine, that are recommended by the International Society for Clinical Densitometry, as the most accurate and reproducible sites in children to assess the BMD [60]. Total body, leg and trunk lean mass, and total fat mass will be derived from the total body DXA scan. The BMD and BMC of total body less head (TBLH) and lumbar spine (L1-L4) results will be presented and analyzed in real values (derived from the machine) and height-adjusted values (uses Lunar international reference database [61]). The necessity of presenting height-adjusted values in the pediatric population has been widely discussed and recognized as appropriate given the impact of short/ tall stature on the densitometry results.

### Physical activity

An activPAL™ accelerometer (PAL Technologies Ltd., Glasgow, UK) will be used to assess participants’ free-living physical activity over 5 consecutive days following their assessment [62]. A 5-day monitoring period was chosen following Ishikawa et al.’s (2013) recommendations, who validated activPAL™ as a reliable measurement tool for children with CP GMFCS levels I–III age 2–14 years old [63]. ActivPAL™ will be attached to the mid-thigh of the dominant leg using a waterproofed dressing. A member of the research team will follow up with families via phone or email in 2 days (36–48 h) after the assessment visit to ensure there are no concerns or issues with the device being used. The average number of steps per day, as well as time of sitting/lying/stepping activities, will be analysed.

### Quality-of-life evaluation

Parents/caregivers will be asked to complete the Cerebral Palsy Quality of Life Questionnaire for Primary Caregiver (CP QOL) to evaluate child social and school well-being, feelings about functioning, pain and impact of disability, general well-being and participation, communication and physical health as well as family health (66 items in total). The questionnaire has shown strong psychometric properties [64] and is widely used for research purposes [16, 65]. To avoid the different perception of the child’s well-being by parents/caregivers, the same person will be asked to fill the questionnaire throughout the duration of the study. The total score and score for each domain will be calculated and analyzed.

### Data collection and management

All data collected will remain confidential. Each participant will be entitled with a unique code and all data and information will be subsequently coded. Only researches will be able to match code with a subject. All collected data and information will be stored in locked cabinets and in a secure computer database which will be accessed only by the study investigators with a password.

Data will be stored in common data formats (.pdf, .txt, .csv) on a secure server for a minimum of 10 years. Hard copies of all assessments and personal information will be stored in a locked cabinet at the Liggins Institute. Scientific data will be used in scientific publications, presentations, and the student PhD thesis. No person will be identifiable from the analysis and publications.

### Safety monitoring process

To monitor safety, the study’s internal safety monitoring committee has been created. The committee will have meetings on a quarterly basis. In case of a serious adverse event(s), the committee will have meetings more frequently. Continued communication with patients/families during the intervention period will assist in identifying adverse events. Participants and their families will be asked to record and immediately report any adverse events that may be associated with VT, including tiredness or pain. All adverse events will be reported to and discussed by the safety monitoring committee.

Vibration therapy will be discontinued in any participant who experiences excessive or persistent pain/aching; experiences bone fractures or any illness that would preclude training. The study will be terminated by the committee if two serious adverse events of the same nature will be registered.

### Statistical considerations

#### Sample size

The sample size calculation was performed in GPower software version 3.1.9.2. For the calculation, we used the mean and standard deviation obtained in the 6MWT from the clinical trial performed of 40 participants with cerebral palsy [14]. Setting the level of statistical significance at 0.05 and power at 0.95, a minimum size of 44 participants for two groups was found. Considering a 10% drop-out rate, the final sample will include 48 participants in total, with 24 for each intervention group.

#### Data analyses

The data will be entered into an Excel workbook on password-protected computers, and later exported into SPSS v25 (IBM Corp, Armonk, NY, USA) and SAS v9.4 (SAS Institute, Cary, NC, USA) for statistical analyses. Baseline demographic and clinical characteristics of participants will be summarised by trial group allocation. Treatment evaluation of the primary outcome (i.e. mobility or distance covered during the 6-min walk test) will be performed on the principle of intention to treat, using data collected from all randomised participants. Data will be analysed using random effects mixed models with repeated measures, to account for the non-independence of measurements on the same participant. Models will incorporate as fixed effects the trial group allocation (20 vs 25 Hz), time-point (assessment II, III, or IV), and their interactions as fixed effects, as well as the participant’s GMFCS level; in addition, the baseline value of the outcome variable will be included as a covariate. There will be no imputation of missing data, as based on our previous studies, we expect very limited or no loss to follow-up. Nonetheless, for a small subset of secondary outcomes, such as certain functional tests on the Leonardo platform that cannot be performed by participants with GMFCS level III, only subgroup analyses will be carried out including solely participants with GMFCS level I-II. Model-adjusted differences between randomization groups and within groups (including the potential effects of treatment duration) will be calculated and reported with 95% confidence intervals. All statistical tests will be two-sided at *p* < 0.05, without adjustments for multiple comparisons.

## Discussion

This clinical trial protocol has several characteristics that distinguish it from previous studies investigating the effectiveness of side-alternating VT in children with CP. Firstly, it evaluates more physical capabilities and health assessment than previous studies including the testing of mobility, body composition, muscle function, gross motor function, respiratory function, and quality of life. To our knowledge, this is the first study that conducts such a comprehensive analysis of the VT effect in children with CP in the age group of 5–12 years old. This approach advances the understanding of the side-to-side alternating vibration training as a therapeutic tool in young children with CP.

Secondly, our study compares the effectiveness of two different VT frequencies: 20 Hz vs 25 Hz. Previous long-term studies on the side-alternation VT applied vibration at different levels of target frequency that varied from 18 Hz to 25 Hz [15, 16, 21, 23, 26]. While the effect of different VT frequencies has been discussed in the literature, no studies have compared the impact of them on children with CP in the same protocol of assessments [24].

Lastly, the study will allow us to compare the effect of short - term (12 weeks) and long-term (20 weeks) VT in children with CP. Twenty weeks intervention period was chosen as a minimal optimal period to promote changes in muscle and bone mass, which was demonstrated in a previously published study by Gusso et al. [16]. Comparing the effects of short-term VT with long-term VT will help us better understand to what extent the short-term VT exposure could promote and optimize outcome parameters or a more prolonged intervention period is required.

The main limitation of the study is the lack of a control group. We decided to utilize a study design where each participant acts as their own control for a number of reasons. Foremost, children with CP present a heterogeneous population with a variety of symptoms, which makes randomization with control and intervention groups problematic [66] and using each child as their own control adjusts for these issues. In this study, each participant serves as his/her own control, with the control period followed by an intervention period. This design has additional advantages. First, it eliminates the variability on the VT response due to participants’ individual characteristics because the statistical analysis is based on the within-subject comparison. Second, it is suitable for the CP population as their symptoms tend to be stable over time [1].

Another anticipated limitation of the study is that the number of the participants who have completed intended assessments may be uneven due to some subjects’ inability to perform some tests (e.g., S2LJT, chair rising test). However, we do not expect this to be a serious limitation, because, according to the eligibility criteria, children with a high level of disability will not be enrolled in the study.

In summary, the proposed study will expand the knowledge on the efficacy and effectiveness of side-alternating VT in children ages 5 to 12 years with mild to moderate CP. It will help understand the benefits and applicability of vibration therapy to improve the muscle and bone health, mobility, and other health aspects. As a result, the study will assist in defining more robust guidelines for the use of vibration therapy in young children with CP and will provide actionable insights to physiotherapists, families and the affected individuals into the optimal ways to use this therapeutic approach.

## Data Availability

Not applicable.

## Abbreviations

ANZCTR: Australian New Zealand Clinical Trials Registry
CP: Cerebral palsy
VT: Vibration therapy
ADHB: Auckland District Health Board
GMFCS: Gross Motor Function Classification System
6MWT: 6-min walk test
10MWT: 10-m walk test
RPE: Rated Perceived Exertion
CRT: Chair rising test
S2LJT: Single two-leg jump test
MAS: Modified Ashworth Scale
GMFM: Gross motor function measure
FVC: Forced vital capacity
FEV1: Forced expiratory volume in 1 s
DXA: Dual-energy X-ray absorptiometry
BMD: Bone mineral density
BMC: Bone mineral content
TBLH: Total body less head
CP QOL: Cerebral Palsy Quality of Life Questionnaire
ANOVA: One-way analysis of variance
